# Probing Local Backbone Geometries in Intrinsically Disordered Proteins by Cross-Correlated NMR Relaxation**

**DOI:** 10.1002/anie.201210005

**Published:** 2013-03-20

**Authors:** Jan Stanek, Saurabh Saxena, Leonhard Geist, Robert Konrat, Wiktor Koźmiński

**Affiliations:** Faculty of Chemistry, University of WarsawPasteura 1, 02093 Warsaw (Poland) E-mail: kozmin@chem.uw.edu.pl; Department of Structural and Computational Biology, Max F. Perutz Laboratories, University of ViennaCampus Vienna Biocenter 5, 1030 Vienna (Austria) E-mail: robert.konrat@univie.ac.at

**Keywords:** cross-correlated relaxation, intrinsically disordered proteins, multidimensional NMR, NMR spectroscopy, protein structures

Proteins that lack stable tertiary structures are attracting growing interest because of their involvement in fundamental biological processes.^1^ The traditional conceptual view correlating structures with functions does not seem to grasp the essential properties of these protein families.^2^ A fundamental problem in the structural characterization of intrinsically disordered proteins (IDPs) is the definition of the conformational ensemble sampled by the polypeptide chain in solution. There is growing evidence that the popular dichotomic partitioning into ordered and disordered proteins does not adequately grasp the dynamic conformational ensembles of biological polypeptides and their mutual similarities.^3^ The recently developed *meta*-structure approach provided first insight into the heterogeneity of folding funnels of disordered proteins suggesting that IDPs populate a diverse conformational space of thermally accessible (sub-)states.^3^ Often the interpretation relies on the concept of residual structure. The observation of a (residue-specific) conformational preference and deviation from an idealized random coil devoid of any structural propensity is interpreted as a prevalence of residual structures. Structural preferences predominantly occur on a secondary structure level. Hydrophobic collapse of preformed local structure elements leads to transiently formed conformations with varying degrees of compaction (for example, molten globules, compact conformations with distinct packing of side chains). NMR spectroscopy has not only been developed into a powerful structural biology technique complementing protein X-ray crystallography, but additionally, it offers unique opportunities for structural and dynamical studies of intrinsically disordered/unfolded proteins. Residue-specific information (with atomic resolution) about hydrogen exchange rates,^4^ chemical shifts,^5^ heteronuclear relaxation rates,^6^ and scalar and dipolar couplings^7^ have provided unprecedented insight into the conformational properties of unfolded proteins in solution. Most importantly, unique long-range structural information is obtained using paramagnetic relaxation enhancement (PRE) data.^8^

Cross-correlated NMR relaxation (CCR) has attracted considerable interest in the past as it offers unique possibilities to probe structural dynamics in proteins.^9^ The method is based on correlated fluctuations of relaxation relevant interaction tensors (for example, dipole–dipole interactions, chemical shift anisotropy (CSA), and quadrupolar interactions). To date, a series of NMR pulse sequences exist that quantitatively measures dipole–dipole (DD), dipolar–CSA (D-CSA), and CSA–CSA cross-correlations. Using these methods, valuable information about structurally relevant dihedral angles of the protein backbone have been obtained. For example, intraresidue ^1^H(*i*)–^15^N(*i*)–^13^C′(*i*) dipolar–CSA interference was proposed to determine the orientation of the ^1^H(*i*)–^15^N(*i*) dipole vector in the principal frame of the intraresidue ^13^C′(*i*) CSA tensor and discriminate between type I and type II β turns in proteins.^10^ As IDPs populate Ramachandran space in a rather unique way and substantially sample β-turn (I,II) and polyproline II helical conformations, novel experimental approaches are highly desirable that allow assess to these (non-α-helical, non-β-strand) conformations in IDPs. Applications of multidimensional (triple-resonance) NMR spectroscopy to IDPs are challenging because of the limited signal dispersion owing to the lack of stably formed tertiary structures. These experimental limitations have been largely overcome by the introduction of higher-dimensionality experiments combined with non-uniform sampling. Herein, we extend the cross-correlated relaxation method to highly crowded NMR spectra typically encountered for IDPs. The novel experiment allows for the quantification of intra-residue ^1^H(*i*)–^15^N(*i*)–^13^C′(*i*) dipolar–CSA interference terms in IDPs exploiting the outstanding spectral resolution of higher-dimensionality NMR experiments.

An outline of the experiment is given in Equation (1). A timing diagram and experimental details of the pulse sequence can be found in the Supporting Information. Briefly, the coherence transfer pathway is as follows:


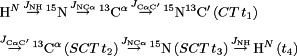
(1)

where ^15^N^13^C′ denotes zero- and double-quantum ^15^N–^13^C′ coherences and CT and SCT are constant and semi-constant time evolution periods, respectively. In terms of the magnetization transfers, the pulse sequence follows the conventional 3D HN(CA)CO experiment^11^ and its four-dimensional extension.^12^ In the CT period, zero- and double-quantum ^15^N–^13^C′ coherences are evolved as suggested previously;^10^ however, only in-phase *J*_NH_-separated doublets are recorded. It is noteworthy that both ^13^C^α^ and ^15^N spins are evolved in the semi-constant time manner for sensitivity enhancement. Interresidual peaks which provide less structural information are usually of smaller intensity due to weaker two-bond coupling ^2^*J*_CαN_≍4–7 Hz.

As a first application of the new pulse sequence, we provide CCR data for BASP1 (270 a.a. chicken brain acid soluble protein 1) at different pH values. Transformation of chicken embryonic fibroblasts by the protein product (c-Myc) of the protooncogene *c-myc* leads to the suppression of BASP1. Strikingly, ectopic expression of BASP1 renders fibroblasts resistant to subsequent cell transformation by c-Myc.^13^ It has been described to be involved in neurite outgrowth and plasma membrane organization, and non-myristoylated BASP1 has been discovered as a co-suppressor of WT1 function (Wilms’ tumor suppressor protein 1).^13^ NMR data obtained for human BASP1 indicate that BASP1 is lacking a well-defined tertiary structure in solution and displays a biophysical behavior reminiscent of an IDP.^14^

Despite extensive spectral crowding in 2D ^15^N-HSQC spectrum, presence of both intra- and interresidual peaks, and *J*_NH_ splitting of each resonance, it was possible to determine the CCR rate for about 82 % of BASP1 residues with amide protons in slow exchange regime at both pH 2 and 6. Clearly, the superior resolution of the four-dimensional spectrum allows data to be extracted without the need to record and combine both in-phase and anti-phase *J*_NH_-modulated data sets.^10^ The closer inspection of the spectra revealed that most of spectral crowding results from overlap of intra- and interresidual resonances, that would not be eliminated by the separation of upfield and downfield doublet lines. Interestingly, error analysis of Γ_H(*i*)N(*i*),C′(*i*)_ (given by Eq. (1) in Ref. 10) leads to the conclusion that optimal precision of cross-correlated relaxation rate is obtained for CT duration comparable to mean *T*_2_ of zero- and double-quantum ^15^N–^13^C′ coherences. Owing to the favorable relaxation properties of intrinsically disordered proteins, CT lengths can be as large as the *T_C_*=90 ms used herein. Clearly, the full utilization of this extraordinarily long constant-time period can only be accomplished using non-uniform sampling. It should be emphasized that the data sets were collected using as few as only 0.24 % of sampling points required conventionally. Very-high-resolution spectra were obtained by employing the signal separation algorithm (SSA) that is known to faithfully reproduce relative signal intensities.^15^ Recently, consistent findings were reported by Coggins and co-workers for a very similar algorithm called SCRUB.^16^ Application of SSA has led to virtually complete removal of sampling artifacts and recovery of natural sensitivity of the experiments. In comparison to zero-augmented Fourier transformation, the average signal-to-noise ratio measured for all relevant intraresidue resonances increased by a factor of 2.66 and 1.31 for BASP1 at pH 6 and pH 2, respectively. Different gain in S/N reflects intrinsic sensitivity associated with various sample concentrations.

As shown with representative C′–C^α^ planes for Thr108 on the Figure 1, inclusion of an extra ^13^C^α^ evolution period to the original 3D experiment greatly enhances the spectral resolution even if ^13^C^α^ chemical shifts in IDPs tend to cluster in the regions specific for a given amino acid type. Seemingly, this is due to the fact that ^13^C^α^ is barely *correlated* to ^13^C′, ^15^N, and ^1^H^N^ chemical shifts.

**Figure 1:**
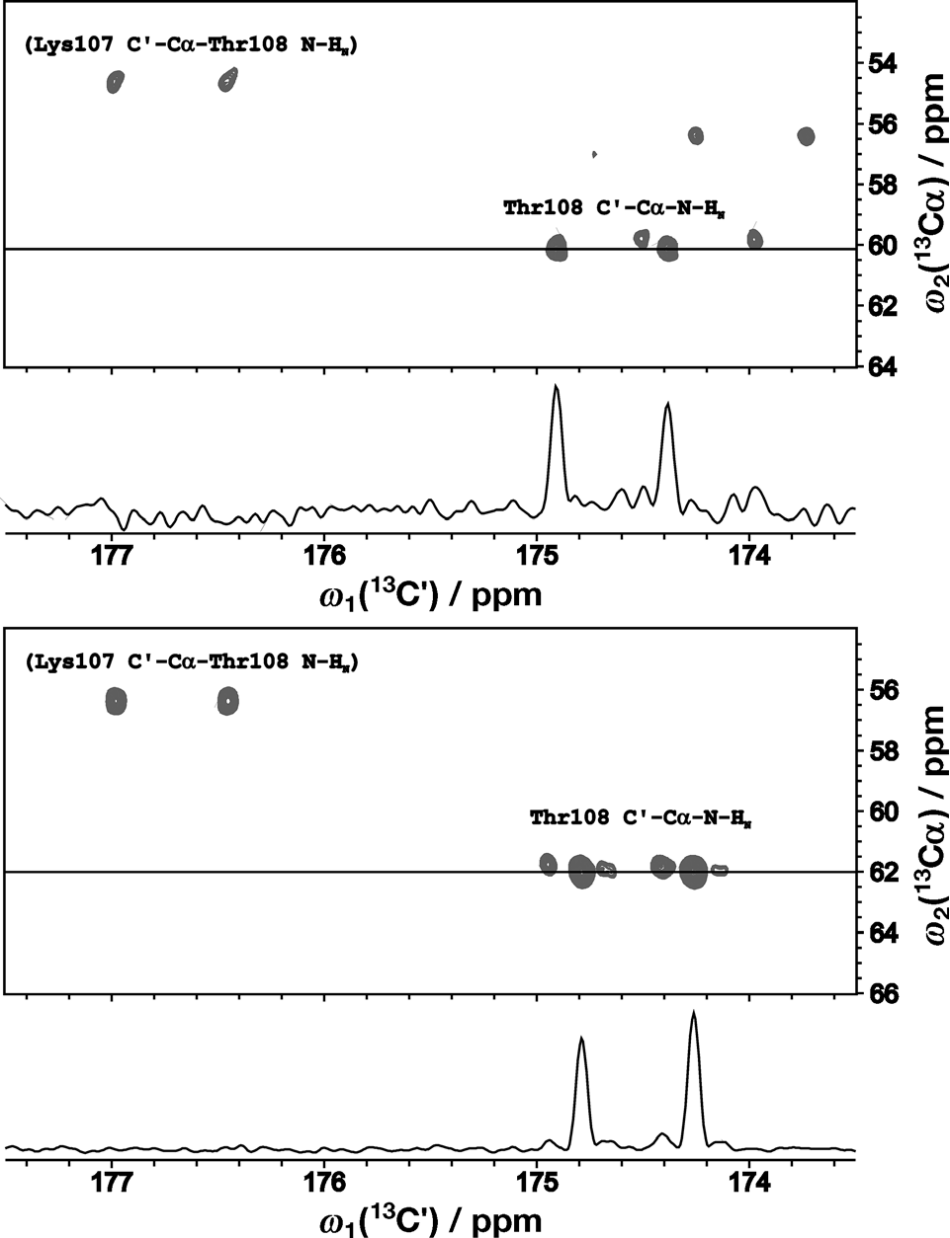
F_1_ (C′)–F_2_ (C^α^) planes from the 4D HNCACO-CCR(C′/NH_N_) spectrum of cBASP1 at pH 2 (top) and pH 6 (bottom) showing *J*_NH_-resolved doublets for threonine 108 residue. Labels for interresidual cross-peaks (irrelevant here) are given in parentheses. Noteworthy is the significant difference in relative intensity of the doublet lines.

Of particular relevance is the fact that intraresidue ^1^H(*i*)–^15^N(*i*)–^13^C′(*i*) dipolar–CSA cross-correlation rates have sensitive dependencies on local β-turn geometries.^10^ While slightly positive values are found in the case of type I β turns, alternating signs of cross-correlation rates are observed for the central residues in a type II β turn (structural information available from this experiment is further discussed in the Supporting Information).^10^ For BASP1 at pH 6, predominantly positive cross-correlation rates were found. In contrast, at low pH (pH 2) significant changes and alternating signs of the cross-correlation rates were found (Figure 2). As can be seen, the changes are distributed along the entire backbone. A closer inspection revealed significant variation of CCR rate for residues 70–80. In the surrounding region acidic amino acids (Glu, Asp) are especially abundant and showed most pronounced chemical shift changes upon lowering the pH (Supporting Information, Figure S2). We thus conclude that the observed cross-correlation rate changes indeed reflect local structural changes induced by protonation of side-chain carboxylic groups. Additionally, changes in the average overall structural ensemble (for example, spatial extension of the conformational ensemble) was probed by DOSY-based measurements of the hydrodynamic radius. Normalized to dioxan, the hydrodynamic radius dropped from 42 Å at pH 6 to 27 Å at pH 2. Furthermore, this compaction was independently verified by SOFAST-HSQC experiments under saturation of aliphatic side-chain protons. Details of this overall structural change is beyond the scope of this current work and will be published in due course elsewhere. Suffice to say here that the pH-induced changes in β-turn geometries can be efficiently detected by the novel NMR approach, suggesting that: 1) subtleties of local backbone structures in IDPs can be assessed using this technique; and that 2) in IDPs a subtle interplay exists between local β-turn geometries and the average extension of the conformational space accessible under changing environmental conditions.

**Figure 2:**
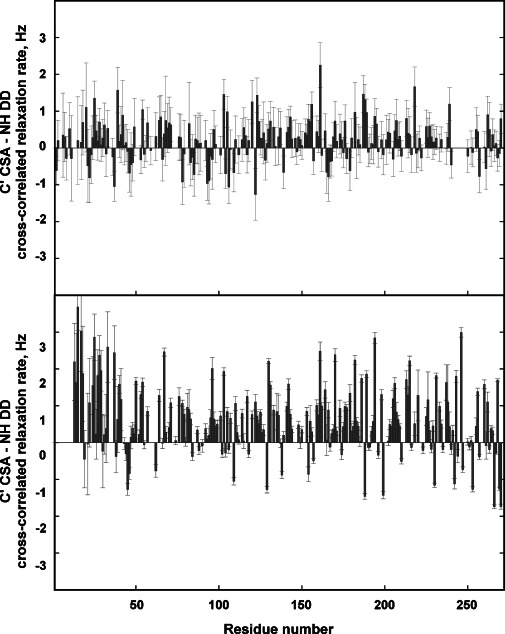
C′ CSA–NH DD cross-correlated relaxation rates for individual residues of BASP1 at pH 2 (top) and pH 6 (bottom).

In summary, we have presented a novel NMR pulse sequence for probing local backbone structure in IDPs. Employing high dimensionality and non-uniform sampling, the novel method allows for the quantification of intraresidue ^1^H(*i*)–^15^N(*i*)–^13^C′(*i*) dipolar–CSA interference in highly overlapped NMR spectra of IDPs. Data obtained on the IDP BASP1 at different pH values illustrated the applicability of the approach to elucidate subtle changes in local backbone geometry under different environmental conditions. Specifically, it was found that in the case of BASP1, lowering the pH induces a population shift of β-turn geometries; at neutral pH, BASP1 predominantly samples type I β turns and polyproline II helices, lowering the pH leads to an increased population of type II β turns accompanied by an unexpected overall compaction of the BASP1 structural ensemble. Given the outstanding sensitivity and spectral resolution of the NMR approach, we envisage useful applications for the characterization of the structural dynamics of IDPs in solution. The detailed data obtained in this study has also provided compelling evidence that intrinsically disordered proteins are characterized by extremely heterogenous energy landscapes, allowing for subtle conformational transitions under changing environmental conditions. Thus, to fully grasp the richness of the structural dynamics of these proteins and their potential role in biological processes, more sophisticated experimental methods as well as theoretical concepts surmounting the conventional order–disorder partitioning are required.

## Supplementary material

As a service to our authors and readers, this journal provides supporting information supplied by the authors. Such materials are peer reviewed and may be re-organized for online delivery, but are not copy-edited or typeset. Technical support issues arising from supporting information (other than missing files) should be addressed to the authors.
