# Supplementary material for: Probing Local Backbone Geometries in Intrinsically Disordered Proteins by Cross-Correlated NMR Relaxation
Source: Angew Chem Int Ed Engl. 2013 Mar 20;52(17):4604–6. doi: 10.1002/anie.201210005 (PMC3659411; doi:10.1002/anie.201210005)
Supplement: Supplementary file 1 [file anie0052-4604-SD1.pdf]

Angewandte Chemie
Eine Zeitschrift der Gesellschaft Deutscher Chemiker

Supporting Information

© Wiley-VCH 2013

69451 Weinheim, Germany

Probing Local Backbone Geometries in Intrinsically Disordered Proteins by Cross-Correlated NMR Relaxation**

Jan Stanek, Saurabh Saxena, Leonhard Geist, Robert Konrat, and Wiktor Koźmiński**

anie_201210005_sm_miscellaneous_information.pdf

Supporting Information

Appendix. Structural information embedded in C`(CSA)-NH(DD) cross-correlation rate

Carbonyl ^{13}C` chemical shift anisotropy – dipolar NH cross-correlation rate is given by

$$\Gamma_{H_N N(i),C`(i)} = \frac{4}{15}\frac{h}{2\pi}\frac{\gamma_H \gamma_C \omega_C}{r_{NH}^{3}}\tau_C \cdot f(\sigma_x,\sigma_y,\sigma_z) \quad [1]$$

where τ_C is the (local) correlation time, γ_H and γ_C are gyromagnetic ratios of ^{1}H and ^{13}C, respectively, r_{NH} is the distance between amide ^{1}H and ^{15}N spins, and ω_C is the Larmor frequency of ^{13}C spins. $f(\sigma_x, \sigma_y, \sigma_z)$ is the determined by magnitude (i. e. principal values) of ^{13}C` CSA tensor and orientation of NH vector in the frame of its principal axes. Thus, this term is also a function of dihedral angles φ_i and ψ_i.

$$f = f(\sigma_x,\sigma_y,\sigma_z,\varphi,\psi) \quad [2]$$

Therefore, a single C`(CSA)-NH(DD) CCR rate does not allow to unambiguously determine these dihedral angles. However, Kloiber and Konrat showed that rather small negative values are characteristic for residues in α-helical regions while greater absolute values are typical for residues in loops [1]. Furthermore, β-turns of type I and II can be distinguished by inspection whether CCR value is nonnegative (type I) or of alternating sign (type II) for consecutive i+1 and i+2 residues in the β-turn.

For example, let us recall the selected CCR values measured for BASP1 at pH=2 and 6:

Residue	pH 2		pH 6	
	CCR, Hz	Error, Hz	CCR, Hz	Error, Hz
Thr76	0.30	0.63	1.25	0.24
Val78	-0.92	0.61	1.06	0.09
Lys79	-0.16	0.91	0.77	0.14
Asn82	0.66	1.12	0.89	0.55
Lys83	-0.40	0.63	0.64	0.16
Glu84	-0.25	0.61	-0.38	0.11
Gln92	0.20	0.55	0.38	0.13
Val93	-0.96	0.46	0.10	0.07
Ser94	-0.89	0.63	0.20	0.19
Ala95	-0.22	0.62	0.67	0.22
Asn96	-0.31	0.81	2.01	0.30
Lys97	0.50	0.52	0.64	0.19
Thr98	0.10	0.50	0.45	0.11

It can be concluded that CCR values are systematically and, for some residues, noticeably smaller at lower pH. This indicates the shift of secondary structure populations towards α-helices upon decrease of pH.

It should be emphasized that variations of local correlation time and anisotropic local motions in the protein backbone influence cross-correlation rates and complicates the quantitative analysis of the results. However, as we have already shown [2] the simultaneous fitting of several backbone-dependent cross-correlation rates lead to reliable distributions of dihedral backbone angles even in the presence of internal mobility. The here proposed multi-dimensional CCR experiment offers additional (independent) information about dihedral angle and further constraints the number of solutions. We thus propose to include this novel CCR experiment in the already described Z-surface approach for backbone dihedral angle determination.

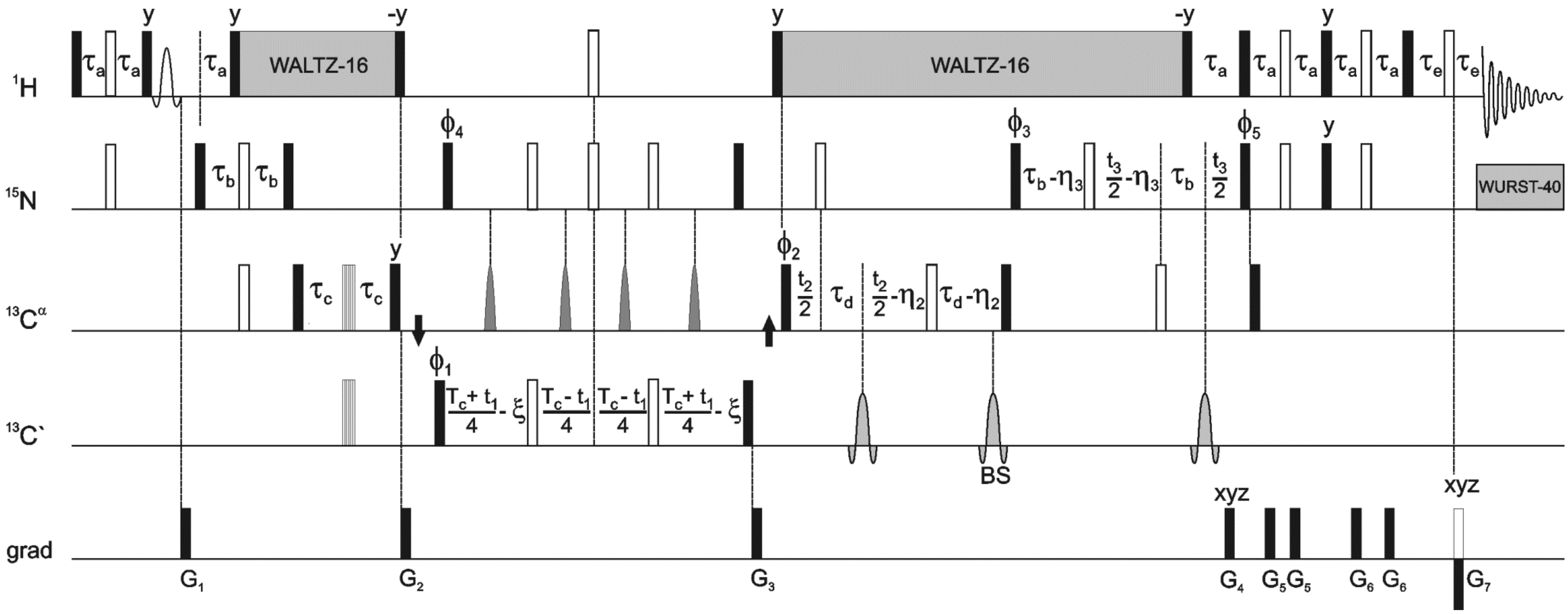

Figure S1. Pulse sequence for the 4D HNCACO-CCR experiment. Narrow and wide bars represent/indicate 'hard' 90° and 180° pulses, respectively. All pulses are applied along the *x*-axis of the rotating frame unless indicated otherwise. ^{1}H and ^{15}N composite pulse decoupling is performed with WALTZ-16 [3] and WURST-40 [4], respectively. Water-selective *sinc*-shaped $\pi/2$-pulse of duration of 1.22 ms is employed. Selective *sinc*-shaped π pulses, with $\gamma B_1/2\pi$ = 12.0 kHz and duration of 68.2μs (adjusted to obtain inversion of C' spin with no effect at C^α) are represented by wide grey *sinc*-shaped pulses. Gray bell-shaped pulses represent $^{13}C^\alpha$-selective Q3 pulses [5] of duration of 220 μs and peak r.f. 14.5 kHz. Six-element composite pulse [6] is employed for simultaneous inversion of C^α and C' spins. Other carbon 90° (180°) pulses are rectangular, with rf. field strength adjusted to $|\Delta\Omega_{CA\text{-}CO}|/\sqrt{15}$ ($\sqrt{3}$) and duration of 46.6μs (41.7μs). Off-resonance pulses were applied using phase modulation of the carrier. 'BS' denotes Bloch-Siegert compensating pulse. The delays are τ_a = 2.69 ms, τ_b = 14 ms, τ_c = 3.15 ms, τ_d = 4 ms, τ_e = 0.35 ms. Constant-time duration T_C is 90 ms. The delay $\xi = 2pw90(C')/\pi$ compensates C' evolution during $\pi/2$ pulses flanking constant-time period. C^α and ^{15}N are evolved in the semi-constant time manner with contraction delays $\eta_2 = \tau_d \cdot t_2 / t_{2,max}$ and $\eta_3 = \tau_b \cdot t_3 / t_{3,max}$. Water magnetization is stored along z-axis for detection to efficiently suppress solvent signal and avoid saturation of amide protons. Quadrature detection in t_1 and t_2 is accomplished by altering ϕ_1 and ϕ_2, respectively, according to the States-TPPI procedure. Echo and anti-echo signals in t_3 dimension were recorded in the interleaved fashion by inversion of gradient G_7 and shift of ϕ_5 by π accordingly. The ϕ_3 and receiver phase are inverted for even numbered points in t_3 to achieve axial peak displacement in ω_3. The phase cycle employed is: $\phi_1 = x, -x$; $\phi_2 = x$; $\phi_3 = x$; $\phi_4 = 2(x),$

2(-x); $\phi_5 = x$; $\phi_{rec} = x, -x, -x, x$. The ^{1}H carrier frequency is set on resonance with the water signal (4.77 ppm). The ^{13}C carrier frequency, initially set to 58.6 ppm ($^{13}C^{\alpha}$), is switched to 176.6 ppm (^{13}C') for the duration of CT block as indicated by vertical arrows. The ^{15}N carrier is placed at 117.8 ppm. Gradients durations and strengths are: G_1 (0.5 ms, 19.5 G/cm), G_2 (1 ms, 14.2 G/cm), G_3 (0.5 ms, 6.4 G/cm), G_4 (2 ms, 31.9 G/cm), G_5 (0.5 ms, 3.5 G/cm), G_6 (0.5 ms, 5.3 G/cm), G_7 (0.2 ms, –/+32.3 G/cm).

Inter-scan delay of 1.2 s was used. 3350 (5000) sampling points (t_1,t_2,t_3) were randomly chosen from 180 × 62 × 125 Cartesian grid according to Gaussian probability distribution $p(t) = \exp[-(t/t_{max})^2/2\sigma^2]$; σ=0.5. The total experiment duration was 44 and 66 h for Chicken BASP1 samples at pH 2 and 6, respectively. Maximum evolution times of 90 (t_1), 10 (t_2) and 50 ms (t_3) were achieved in the indirectly detected dimensions. Spectral widths of 2.0 (ω_1), 6.2 (ω_2), 2.5 (ω_3) and 12 kHz (ω_4) were assumed.

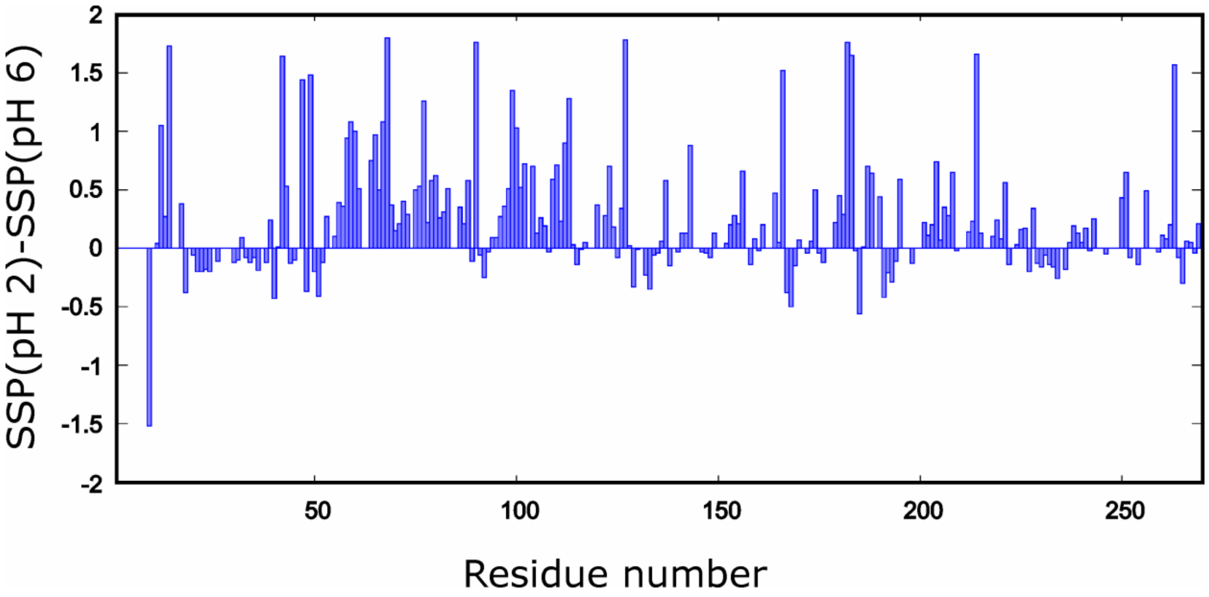

Figure S2. Change of secondary structure propensity (SSP) of BASP1 upon pH drop from 6 to 2 plotted for individual aminoacid residues across full protein length. SSP for a particular pH was calculated as a difference of C^{α} and C^{β} chemical shifts (δC^{α}-δC^{β}). Noteworthy is the significant change of SSP for residues 30-120 indicating increased preference for helical conformations at lower pH.

References:

[1] K. Kloiber, R. Konrat, *J. Am. Chem. Soc.* **2000**, *122*, 12033-12034.
[2] K. Kloiber, W. Schüler, R. Konrat, *J. Biomol. NMR* **2002**, *22*, 349-363.
[3] A. J. Shaka, J. Keeler, R. Freeman, *J. Magn. Reson.* **1983**, *53*, 313-340.
[4] Ē. Kupče, R. Freeman, *J. Magn. Reson. Ser. A* **1995**, *115*, 273-276.
[5] L. Emsley, G. Bodenhausen, *J. Magn. Reson.* **1992**, *97*, 135-148.
[6] A. J. Shaka, *Chem Phys Lett* **1985**, *120*, 201-205.
